# Mental Health Determinants Among a Psychiatric Outpatient Sample of Vietnamese Migrants in Germany

**DOI:** 10.3389/fpsyt.2020.580103

**Published:** 2020-12-23

**Authors:** Simon Wolf, Eric Hahn, Katja Wingenfeld, Main Huong Nguyen, Anita von Poser, Thi Hoa Nguyen, Bernd Hanewald, Kerem Böge, Malek Bajbouj, Michael Dettling, Van Tuan Nguyen, Thi Minh Tam Ta

**Affiliations:** ^1^Department of Psychiatry and Psychotherapy, Charité–Universitätsmedizin Berlin, Berlin, Germany; ^2^Institute of Social and Cultural Anthropology, Freie Universität Berlin, Berlin, Germany; ^3^Department of Psychiatry, Hanoi Medical University, Hanoi, Vietnam; ^4^Department of Psychiatry and Psychotherapy, Universitätsklinikum Gießen and Marburg, Gießen, Germany; ^5^Berlin Institute of Health, Berlin, Germany

**Keywords:** Vietnamese, Germany, migrants, BSI-18, mental health determinants

## Abstract

**Background:** Mental health risk-factors for Asian migrants have been studied almost exclusively in the US, Canada, and Australia but not in European countries. Therefore, we aimed to identify sociodemographic, clinical, and migration-surrounding factors associated with experienced mental distress among Vietnamese migrants in Germany.

**Method:** 305 Vietnamese migrants utilizing Germany's first Vietnamese psychiatric outpatient clinic filled out at admission the Brief-Symptom-Inventory 18 (BSI-18) as well as a questionnaire on 22 potential mental health determinants. Using a multiple linear regression model, we identified those sociodemographic, clinical, and migration-surrounding factors that were significantly related to the Global Severity Index (GSI) of the BSI-18.

**Results:** The factors *unemployment* (*B* = −6.32, *p* = 0.014), *financial problems* (*B* = −10.71, *p* < 0.001), *no or only little religious involvement* (*B* = −3.23, *p* = 0.002), *no psychiatric precontact* (*B* = −7.35, *p* = 0.004), *previous migration experiences* (*B* = 8.76, *p* = 0.002), and *perceived discrimination* (*B* = 6.58, *p* = 0.011) were found to significantly increase the level of mental distress according to the BSI-GSI.

**Conclusion:** Based on these results, we were able to construct a mental health risk-profile for Vietnamese migrants in Germany, which aims to detect candidates for psychiatric problems earlier and supply them with customized prevention and therapy options.

## Introduction

Even today, Asian migrants are still being found to underreport psychological complaints and to under utilize mental health care services worldwide (1). Therefore, gaining improved clinical access to these “hard-to-reach” populations represents an ongoing challenge for the mental health care system of the twenty-first century. An essential step forward might be the early diagnostic identification and supply of candidates for psychiatric problems by implementing risk-profiles and adapted therapy options. At the same time, however, preliminary research on the specific components of such risk-profiles remains to be rare and widely restricted to Asian migrants in the US, Canada, and Australia.

With regard to Asian Americans, Kramer et al. (2) formulated eight “key factors” of particular interest when dealing with their mental health situation: *age*; *gender*; *language skills*; *occupation-related issues*; *religiosity and spirituality*; *traditional beliefs about mental health*; *the level of acculturation;* and *family or intergenerational issues*. Additionally, the authors noted that the *socioeconomic condition* and *immigration status* play a significant role for the mental health and well-being of Asian migrants (2). A large body of empirical research has confirmed the relevance of these key factors. The factor *age* was found to impact mental health in two ways: Asian Americans with a younger age at migration exhibited a higher 12-month and lifetime risk for mental disorders (3). Furthermore, advanced age was associated with increased psychiatric problems among older Asians in Canada (4). The factor *gender* appears to affect mental health differently depending on the type of psychiatric disorder taken into consideration. While significantly more mood- and anxiety-related complaints were reported among South Asian women in Canada (5), the opposite was the case concerning the rates of substance abuse among Indians in the United States (6). However, it should be noted, that this finding does not only account for Asian men and women in the US or Canada, but also for the mainstream populations worldwide, where mood- and anxiety-disorders appear to be generally more widespread among women. In contrast, addiction-disorders seem to be more common among men (7). The lack of sufficient *language skills* has been identified as an independent mental risk-factor, for example, among Southeast Asians in Canada (8), but also as an aggravating factor when hindering American Asians from utilizing treatment due to perceived language barriers (9). The same applies to the factor *unemployment*, as it was revealed to directly reduce the mental health status of Southeast Asians in Canada (8), while simultaneously exasperating other risk factors, such as an already *strained financial situation* linked to the migration process or educational disadvantages and social exclusion (8). *Religious involvement* and *mental health beliefs* are often interrelated in their impact on mental health, especially among Asians, due to the close connection between religion and health within their value system (10). Accordingly, studies have documented a positive effect of religiosity on mental health, for instance, higher self-rated subjective well-being among Chinese Americans with more frequent religious attendance (11). Some studies, however, have reported the opposite when religiosity interferes with the concept of mental health (care) (12).

Regarding the factor *level of acculturation*, studies among Vietnamese migrants in Germany have linked both, the orientation toward the heritage society as well as toward the mainstream society to a decreased level of depressiveness, while the rejection of both (in the form of the marginalization acculturation strategy) was found to be associated with the highest severity level of depression (13). Furthermore, the pattern of mental health care utilization appears to depend on the level of acculturation, as has been shown in a qualitative survey of Asian migrants in Australia (14). Finally, *family and intergenerational issues* can negatively affect Asian migrants' mental health through a gap between traditional Asian family values and those of the mainstream society (15).

In contrast to the number of studies on mental health determinants of Asian migrants in the US, Canada, and Australia, risk-factors for the mental health of European Asians have been studied very little so far and only in conjunction with other migrant populations but not individually (16). One of these barely researched groups are Vietnamese migrants in Germany—with around 99,000 individuals in 2019 one of the country's largest Asian populations, and with 26,000 members the largest Southeast Asian community in the capital of Berlin (17). Among the few existing German studies on this migrant group, a survey of 82 Vietnamese living in the city of Leipzig exhibited higher anxiety and depression scores as well as a lower mental health care utilization than in the German control sample (18). Moreover, social assimilation and perceived discrimination were identified as significant influencers of both psychiatric syndromes. These results were partly confirmed by subsequent studies when the number of experienced migration-associated stressors (including discrimination) (19), as well as the strategy of acculturation (13), were found to impact the severity of depression among Vietnamese outpatients in Berlin.

Apart from these very few studies, however, there have been hardly any further investigations on mental health determinants among Vietnamese migrants in Germany. Therefore, using data from a Vietnamese psychiatric outpatient-cohort, the present study aimed to identify sociodemographic, clinical, and migration-related risk-factors associated with experienced psychological distress, as indicated by the Global Severity Index of the BSI-18. The resulting profile could then be used to detect persons at risk earlier and supply them with adapted prevention and therapy options.

## Methods

### Procedure

The data was collected between 2012 and 2018 within the specialized psychiatric outpatient clinic for Vietnamese migrants at the Charité - Universitätsmedizin, Department for Psychiatry and Psychotherapy, Campus Benjamin Franklin, Berlin, Germany. All patients attending the clinic were initially screened with the Mini International Neuropsychiatric Interview [MINI 5.0 (20)] by trained bilingual psychiatrists.

Subsequently, those patients fulfilling the criterion of having at least one psychiatric diagnosis according to ICD-10 answered the Brief Symptom Inventory-18 [BSI-18; (21)]. The questionnaire was administered in either the German or the Vietnamese language. Since no Vietnamese version of the BSI-18 has been validated yet, we translated the German Version into written Vietnamese language using the technique of back-translation, carried out by different bilingual translators (22). Afterwards, all patients received another questionnaire in either the German or Vietnamese language, assessing sociodemographic, clinical, or migration-related facts.

Patients with signs of acute suicidality, according to the MINI, were excluded from the study. Data used for the current research was exclusively collected at patients' first admission before undergoing treatment. All subjects gave written informed consent before participating and received no financial compensation. The study was approved by the ethics committee of the Charité - Universitätsmedizin, Berlin, Germany.

### Instruments

The outcome criterion of interest was the Global Severity Index (GSI) of the Brief Symptom Inventory-18. The BSI-18 is a frequently used self-report questionnaire that is designed to measure the current level of psychological distress in adult individuals. It consists of three subscales with each assessing a common psychiatric syndrome, namely somatization (items 1, 4, 7, 10, 13, 16), depression (items 2, 5, 8, 11, 14, 17), and anxiety (items 3, 6, 9, 12, 15, 18). The 18 items are allocated equally across the three subscales, with each examining on a 5-point Likert scale (from 0 = “not at all” to 4 = “extremely”) to what extent the respective symptom burdened the respondent during the previous 7 days. The subscales' raw scores (ranging from 0 to 24) can be interpreted individually or summed up to create the GSI (ranging from 0 to 72), whereby higher ratings reflect considerably more experienced distress. The BSI-18 has been shown to have good psychometric reliability (23). In the current study, the average intercorrelation (Cronbach's α) between all BSI-18-items was α = 0.96, indicating an excellent internal consistency for the BSI-GSI outcome measure.

Potential predictive factors were taken from a questionnaire originally containing a total of 39 items about sociodemographic, clinical, and migration-related factors that was completed by all patients upon the first admission to the outpatient clinic. Based on theoretical considerations as well as on extensive literature research (Medline, PSYNDEX, and PsycINFO) on factors that are generally relevant for the mental health of Asian migrants, we preselected 22 questionnaire-items as potential BSI-GSI predictors. This procedure was chosen because a theoretical framework regarding factors that are especially relevant for the mental health status of Vietnamese migrants has to our knowledge not been published yet.

Sociodemographic candidate variables were *age* (continuous variable), *gender* (binary variable: “male” or “female”), *marital status* (binary variable: “without partnership” or “in a partnership”), *number of children* (continuous variable), *number of household members* (continuous variable), *years of education* (continuous variable)*, occupational status* (binary variable: “in work” or “no work”), *financial status* (binary variable: “not sufficient” or “sufficient”), and *the current role of religion* (continuous variable with a four-point response option ranging from “not important” to “very important”). Clinically relevant candidate variables were the *duration of persisting symptoms* (continuous variable, in months), *precontact to the psychiatry* (binary variable: “no” or “yes”) and *past suicide attempt(s)* (binary variable: “no” or “yes”). Migration-related candidate variables were *years lived in the host country* (continuous variable), *age at migration* (continuous variable), *the status of current residence* (binary variable: “unsafe” or “safe”), *German language skills* (continuous variable with a five-point response option ranging from “very good” to “none”), *previous migration experiences* (binary variable: “no” or “yes”), *feeling of being at home in Germany* (binary variable: “no” or “yes”), *close connection to the home country* (binary variable: “no” or “yes”), *perceived discrimination* (binary variable: “no” or “yes”), and the two dimensions of acculturation: the *identification toward the mainstream society* (continuous variable) as well as the *identification toward the ethnic society* (continuous variable).

The two dimensions of acculturation were assessed with the Stephenson Multigroup Acculturation Scale [SMAS; (24)]. The scale consists of a 15-item Dominant Society Immersion (DSI) subscale that measures participants' identification toward the mainstream society and a 17-item Ethnic Society Immersion (ESI) subscale that measures participants' identification toward their ethnic society. After the participants had rated each item on a 4-point Likert scale (1 = “false,” 2 = “partly false,” 3 = “partly true,” and 4 = “true”), summative scores were computed for each subscale with higher scores indicating a greater level of immersion toward the dominant or ethnic society. In our study Cronbach's α over all 32 SMAS-items was 0.80 as well as 0.85 for the DSI- and 0.79 for the ESI-subscale, demonstrating good internal consistency of the scale.

### Statistical Analysis

Descriptive statistics were used to describe the sample characteristics. Single item scores of all 18 BSI-items were summed up to create the dependent outcome variable BSI-GSI. For every participant, the sum score was only calculated if at least 75% of the BSI-18 items were rated. A drop-out analysis was conducted to assess possible differences in *age, gender* and *level of education* between participants with less and more than 25% missing data on the BSI-18 items. Cronbach's α was calculated as an indicator of internal consistency of the BSI-GSI scale and the SMAS scale. Preliminary analyses were performed to ensure that there was no violation of the assumption of normality, linearity, multicollinearity, and homoscedasticity.

The principal analysis was carried out in two steps: First, to explore the statistical relationship of each candidate variable with the BSI-GSI-score, we conducted three separate multiple regression models, one model with the sociodemographic and the other two with the clinical and migration-related variables. Second, those variables with a *p*-value of ≤ 0.05 were then included in a final multiple linear regression model, to achieve the most informative and parsimonious combination of BSI-GSI predictors. Statistical analyses were calculated with IBM SPSS (Version 24 for macOS). Values of *p* ≤ 0.05 were considered as statistically significant and values of *p* ≤ 0.001 as highly significant.

## Results

### Sample Characteristics

The demographic and migration-related sample characteristics of the in total 305 patients with Vietnamese background are shown in Table 1. Regarding further clinical details, the mean BSI-GSI score was 23.54 (SD = 19.22). Across all diagnostic categories, the symptomatology was present for an average time of 23.0 (SD = 37.7) months at the date of the first admission. 92 (30.2%) patients had a former contact with the mental health care system, and 29 (9.9%) had attempted suicide at least once in the past.

**Table 1:** Sample Characteristics (*N* = 305).

**Characteristics**	***n*^**a**^ (%)**	***M (SD)*^**b**^**
**Sociodemographic characteristics**		
Age (in years)		44.7 (12.9)
Gender (female)	233 (76.4%)	
Marital status (has partner)	132 (43.6%)	
Number of children		1.8 (1.4)
Number of household members		2.6 (1.4)
Years of education		10.0 (3.1)
Occupational status (working)	57 (26.5%)	
Financial status (sufficient)	65 (21.8%)	
Current role of religion		
Not important	44 (14.7%)	
Less important	72 (24.1%)	
Important	50 (16.4%)	
Very important	139 (45.6%)	
**Acculturation characteristics**		
Years lived in the host country		16.2 (10.4)
Age at migration		27.5 (10.1)
Status of current residence (unsecure)	138 (45.7%)	
German language skills		
Very good	11 (3.7%)	
Good	23 (7.7%)	
Moderate	93 (31.1%)	
Few	110 (36.8%)	
None	62 (20.7%)	
Previous migration experiences (yes)	62 (21.1%)	
Feeling of being at home in Germany (yes)	199 (67.5%)	
Close connection to the home country (yes)	217 (73.1%)	
Perceived discrimination (yes)	104 (34.1%)	
Dimension of acculturation		
Dominant Society Immersion (DSI)		33.05 (9.32)
Ethnic Society Immersion (ESI)		56.24 (10.20)
**Clinical characteristics**		
ICD-10 main diagnosis (F5x, F7x and F9x unrepresented)		
F0x	9 (3.0%)	
F1x	2 (0.7%)	
F2x	42 (13.8%)	
F3x	193 (63.3%)	
F4x	56 (18.4%)	
F6x	2 (0.7%)	
F8x	1 (0.3%)	
Average length of symptomatology (in months)		23.0 (37.7)
Precontact to the psychiatry (yes)	92 (30.2%)	
Past suicide attempt (yes)	29 (9.9%)	

a *Subsample (values vary because of missing data)*;

b *Mean (Standard Deviation)*.

### Drop-Out Analysis

The data of 51 participants were not included in the analysis because more than 25% of their BSI-18 information was missing. A drop-out analysis was conducted to assess potential deviations in *age, gender*, and *years of education* between participants with less and more than 25% missing data. However, no significant differences were found between the groups of included and excluded participants, regarding *age* [*t*_(303)_ = −0.899, *p* = 0.369], *gender* [χ^2^_(1, n = 305)_ = 0.502, *p* = 0.479], and *years of education* [*t*_(297)_ = 0.599, *p* = 0.133].

### Sociodemographic, Clinical, and Migration-Related Predictors of the GSI

Out of 22 initially considered candidate variables, the three separate multiple regression models yielded in total six predictors with a *p* ≤ 0.05 (Table 2): *unemployment* (*B* = −5.63, *p* = 0.047), *financial problems* (*B* = −9.85, *p* = 0.002), *no or only little religious involvement* (*B* = −4.31, *p* < 0.001), *no psychiatric precontact* (*B* = −7.19, *p* = 0.009), *previous migration experiences* (*B* = 7.87, *p* = 0.018), and *perceived discrimination* (*B* = 6.53, *p* = 0.025). These variables were then included in a multiple linear regression model, whereby all variables kept statistical significance after applying the enter-method: *unemployment* (*B* = −6.32, *p* = 0.014), *financial problems* (*B* = −10.71, *p* < 0.001), *no or only little religious involvement* (*B* = −3.23, *p* = 0.002), *no psychiatric precontact* (*B* = −7.35, *p* = 0.004), *previous migration experiences* (*B* = 8.76, *p* = 0.002), and *perceived discrimination* (*B* = 6.58, *p* = 0.011). The final model, as displayed in Table 3, accounted for 18.7% of the BSI-GSI variance.

**Table 2:** Results of the three multiple regression models for sociodemographic, clinical, and migration-related predictors of the BSI-18 GSI.

**Predictor**	***B*^**a**^**	**SE^**b**^**	**β^c^**	***t*^**d**^**	***p*^**e**^**	**CI^**f**^**
**Model 1 (sociodemographic factors)**						
Age	−0.113	0.11	−0.072	−0.99	0.337	−0.34; 0.11
Gender	−2.056	3.11	−0.044	−0.66	0.509	−8.19; 4.08
Marital status	1.365	2.73	0.036	0.50	0.618	−4.03; 6.76
Number of children	−0.037	1.13	−0.003	−0.03	0.974	−2.27; 2.20
Number of household members	−1.116	1.14	−0.079	−0.98	0.327	−3.36; 1.13
Years of education	−0.039	0.42	−0.006	−0.09	0.927	−0.88; 0.80
Occupational status	−5.632	2.82	−0.132	−2.00	0.047	−11.18; −0.82
Financial status	−9.848	3.17	−0.198	−3.17	0.002	−16.09; −3.60
Importance of religion	−4.311	1.14	−0.254	−3.77	<0.001	−6.56; −2.06
**Model 2 (clinical factors)**						
Duration of persisting symptoms	0.022	0.03	0.047	0.72	0.475	−0.04; 0.08
Precontact to the psychiatry	−7.194	2.72	−0.175	−2.64	0.009	−12.55; −1.83
Past suicide attempts	4.253	4.41	0.064	0.97	0.336	−4.43; 12.94
**Model 3 (migration-related factors)**						
Age at migration	−0.083	0.14	−0.044	−0.61	0.543	−0.35; 0.18
Years lived in the host country	0.152	0.19	0.082	−0.79	0.432	−0.23; 0.53
Status of current residence	−4.640	2.73	−0.197	−1.99	0.067	−9.53; 0.47
German language skills	2.229	1.40	0.119	1.59	0.112	−0.53; 4.99
Feeling of being at home in Germany	1.846	2.97	0.044	0.62	0.835	−4.01; 7.71
Previous migration experiences	7.874	3.29	0.165	2.39	0.018	1.39; 14.36
Close connection to the home country	0.818	1.58	0.035	0.52	0.605	−2.29; 3.94
Perceived discrimination	6.534	2.89	0.160	2.26	0.025	0.84; 12.23
DSI (SMAS)	−0.008	0.19	−0.192	−0.43	0.671	−0.05; 0.03
ESI (SMAS)	0.004	0.19	0.097	0.22	0.829	−0.34; 0.04

a *Unstandardized Beta-coefficient*;

b *Standard error*;

c *Standardized Beta-coefficient*;

d *T-value*;

e *p-value*;

f *Confidence Interval (95%)*.

**Table 3:** Multiple regression analysis for predictors of the BSI-18 GSI-score: final model after pre-analyses.

**Predictor**	***B*^**a**^**	**SE^**b**^**	**β^c^**	***t*^**d**^**	***p*^**e**^**	**CI^**f**^**
Occupational status	−6.322	2.55	−0.150	−2.48	0.014	−11.35; −1.29
Financial status	−10.707	2.85	−0.226	−3.76	<0.001	−16.32; −5.10
Importance of religion	−3.232	1.05	−0.190	−3.09	0.002	−5.29; −1.17
Precontact to the psychiatry	−7.354	2.51	−0.177	−2.93	0.004	−12.29; −2.41
Previous migration experiences	8.756	2.85	0.187	3.08	0.002	3.15; 14.36
Perceived discrimination	6.580	2.56	0.157	2.57	0.011	1.54; 11.62

a *Unstandardized Beta-coefficient*;

d *Standard error*;

d *Standardized Beta-coefficient*;

d *T-value*;

d *p-value*;

d *Confidence Interval (95%)*.

## Discussion

While some data on factors influencing the mental health of Asian migrants in the US, Canada, and Australia have been published, research in this area still lacks for Asians in Europe. Thus, the present study aimed to identify sociodemographic, clinical, and migration-surrounding risk-factors for mental distress among a cohort of Vietnamese migrants, who visited a psychiatric-psychotherapeutic outpatient clinic in the city of Berlin. Out of 22 initially considered candidate variables, the following five were found to be significantly related to the current level of experienced psychological distress: (1) *unemployment*, (2) *financial problems*, (3) *no or only little religious involvement*, (4) *no psychiatric precontact*, (5) *previous migration experiences*, and (6) *perceived discrimination*.

Consistent with studies on other migrant populations (25, 26), the factor *unemployment* was found to increase mental distress among Vietnamese migrants in Germany. On the one hand, unemployment is associated with numerous psychiatric complaints in different populations worldwide, not just in migrants (27). On the other hand, unemployment can be seen as a particular burden for migrants, when hindering them to socially integrate or gain permanent residency in the host country (25). Furthermore, unemployment often aggravates an already strained financial situation linked to the migration process. This, in turn, may produce additional distress since socioeconomic difficulties were reported continuously to cause mental problems in migrant (28) and non-migrant populations (29). Accordingly, the current study found a strong association between experienced *financial difficulties* and increased psychological distress. Especially for those Vietnamese who immigrated to Germany for economic reasons, the experience of ongoing financial pressure and insecurity may lead to a sense of hopelessness and reduced self-efficacy expectation. This condition was revealed to increase the severity of depression in one of our previous studies on this migrant group (19).

In line with studies on other migrant populations reporting a positive relationship between *religiosity* and mental health (30, 31), we found less psychiatric distress among religiously involved Vietnamese in Germany. Possible mechanisms are the evocation of positive emotions, the experience of purpose and meaning, the support from the religious community as well as the stress-buffering role of faith-based optimism. Therefore, in the frequently challenging context of migration, a lack or loss of the religious community may be conceptualized as the absence of an additional source of resilience. At the same time, however, studies have shown that stronger religious beliefs are correlated with negative attitudes toward psychiatry and psychiatrists in India (32) and Vietnam (12) and could, therefore, lead to a reduced utilization of treatment facilities and offers (33). Hence, the positive effect of a psychiatric-psychotherapeutic treatment, as well as its compatibility with religious practices, should be discussed with engaged members of the religious communities (see recommendations in Table 4).

**Table 4:** Stressor-specific clinical recommendations.

**Stressor**	**Clinical recommendation**
Financial problems	• Financial problems may be especially demanding for those Vietnamese who migrated for economic reasons, because they might consider their migration-project to be failed • Clinicians should be able to inform about official offers to get help with economic problems • If currently not directly solvable, coping skills should be strengthened to relieve the patient and facilitate the subsequent treatment Therefore, resource- and empowerment-oriented psychotherapy appears appropriate for Vietnamese patients with socioeconomic difficulties
Unemployment	• Clinicians should be aware that unemployment is often accompanied by other problems, like social exclusion and self-doubts • Migrant-specific offers (language courses and specialized job training) should be pointed out • Occupational rehabilitation, as well as an occupational therapy approach is recommended for Vietnamese psychiatric patients
No or low religious involvement	• Mental health care providers should consider religiosity as a potential resource and include it in the clinical setting • At the same time, they should be aware of potential aggravating interferences between religiosity and the therapeutic process or the intake of medication
No psychiatric precontact	• Clinicians should be aware that even when the severity of mental distress elevates, this does not necessarily mean that the willingness to utilize mental health care services increases likewise • Therefore, at the first admission, the reasons for a potential delay in mental health care use should be thoroughly explored (e.g., concerns about shame and stigma, linguistic inappropriateness of services, cultural shaped concept of mental health which is inconsistent with Western forms of treatment) • Brief anti-stigma interventions should be implemented in order to minimize or even eliminate barriers in the future • Close cooperation between (preferably bilingual and bicultural) mental health care providers and community workers might help to reduce hurdles and to inform about consequences of a delayed help-seeking
Previous migration experiences	• Clinicians should pay attention to the complexity of migration experiences and migration ways • The diagnostic clarification should entitle a detailed history of migration, as well as a screening for pre-, post-, and peri-migration stressors
Perceived discrimination	• Clinicians should be aware that a Vietnamese psychiatric patient could have been the target of multiple discrimination: because of having a migration background *and* because of having mental problems • Clinicians should keep in mind that they have an exemplary role in how to validate and deal with mental problems adequately • Clinicians should be able to inform persons affected about anti-discrimination laws and local anti-discrimination agencies

At first glance, our finding of less severe psychological impairment among Vietnamese with at least one *psychiatric precontact* may appear surprising, because studies on other populations have shown that the likelihood of mental health care utilization increases with the severity of psychiatric problems, such as major depression (34, 35). However, since psychiatric institutions are often negatively perceived among Vietnamese (12), a possible explanation might be, that patients who had no former contact with the mental health care system may seek help only in the case of severe distress, when it is perceived to be no longer manageable. This late utilization of psychiatric services was also found in the present sample, as symptoms lasted on average 23 months at the time of the first admission to the outpatient clinic. Patients with at least one previous contact with the mental health care system, in turn, could attend services earlier because of already having overcome their reluctance and being less afraid to use the respective facilities again. Such earlier access may then prevent the deterioration and chronicity of the symptoms by timely given therapeutic and psychopharmacological interventions.

One possible explanation for our finding of considerable more distress in the case of *previous migration experiences* is the phenomenon that on their way to Germany, many Vietnamese had a migratory “stop-over” in the former Eastern Bloc states (36). This often increased the complexity and duration of their migration process and, therefore, the probability of stressor-exposure. To give an example, Vietnamese migrants with several stops in different countries might be more often and for a longer time confronted with an uncertain residence status and the resulting fear of rejection than those who came to Germany directly (37). Therefore, our results suggest that multiple migration experiences may enlarge the stressor load and, thereby, as previous studies with Vietnamese in Germany (19) and Sweden (38) have shown, the risk for adjustment problems and mental distress such as depression.

Finally, the experience of interpersonal *discrimination* was found to be a major mental health risk-factor. Perceiving unfair treatment toward oneself or one's in-group represents a psychosocial stressor that is frequently related to poor psychological and physical health among migrant and non-migrant minority groups (39). Accordingly, studies on Vietnamese migrants in the US (40) and Europe (18) have documented a general decline in mental health under the influence of discrimination, but have also reported several moderators with the potential to reinforce or mitigate the negative impact of discrimination, such as social support (41) or the generational status (42). In addition, not only the effect of discrimination on mental health but also the perception of discrimination itself can be influenced by several characteristics, such as the current mood state (43) or the role of expectation (44). However, these methodological peculiarities notwithstanding, when dealing with Vietnamese migrants in the clinical setting, healthcare professionals should consider the migration background by screening for potential discriminatory experiences and other psychosocial stressors.

The factors *age, gender, marital status, number of children, number of household members, years of education, duration of persisting symptoms, past suicide attempt(s), age at migration, years lived in the host country, status of current residence, German language skills, feeling of being at home in Germany, close connection to the home country*, and the *two dimensions of acculturation* were not found to be significantly associated with the current level of experienced mental distress among our Vietnamese outpatient sample.

Regarding the factor *age*, most studies on other migrant populations have revealed a mental health advantage for younger (45) and disadvantage for older (46) migrants while the middle-aged frequently appear relatively unaffected. Therefore, concerning our Vietnamese sample, it cannot be entirely determined if age indeed was not a mental health key factor or if it has merely not unfolded its full effect since our participants were, on average, 45 years old and thus not part of any risk group. Regarding the factor *gender*, several studies have revealed a heightened mental health risk for female Vietnamese (45). However, some studies on other migrant populations have reported the opposite; for example, increased PTSD rates among male migrants in Finland (47). This underlies the general importance of this factor but also its variability with changing backgrounds.

The sociodemographic characteristics *marital status, number of children*, and *number of household members* were likewise not found to be among the most influential factors. However, they were revealed as crucial mental health determinants in the literature elsewhere (48). This may seem surprising at first sight since social contacts play an essential role within the Vietnamese value system (49) and their absence should, therefore, be a demanding condition for those being without a partner, children, or household members. However, since our sample was mainly comprised of Vietnamese living in Berlin, a city with a large and vibrant Vietnamese community, the lack of domestic contacts might be buffered or even compensated by the possibility to participate in overarching community networks and activities. Another explanation might be the increased use of social media, which enables migrants to stay in contact with distant friends and relatives, even when being less involved in the local community or living in a single-person household. Therefore, more research is needed to clarify how the role of domestic contacts is moderated by sociogeographical conditions and contact options via social media.

According to previous research, a higher *educational level* is often associated with improved mental health behavior (50). While most of the included Vietnamese patients indeed stated to have an intermediate or high-level Vietnamese school degree, the association mentioned above might not account for them because the concept of mental health (care) is still relatively new in Vietnam and might, therefore, not yet have found its way into the education system. Concerning the factor *language skills*, studies on Vietnamese (51) and other migrant populations (52) have confirmed a direct link between insufficient language proficiency and mental distress as well as an indirect link through a reduced mental healthcare use because of perceived language barriers (9). Although our present sample indeed displays deficient language skills, with over 55% rating their German language skills as “few” or “none,” there was no sustainable effect on their subjective well-being. One reason might be that First Generation Vietnamese migrants in Germany were often found to stay within their communities, rendering a proficiency of German language skills less critical (53). Another reason might be that due to increased psychological distress, some respondents may not have been able to learn the host language properly, although there was a desire to do so.

In contrast to previous research, the present study did not find the migration-related characteristics *status of current residence* and *years lived in the host country* to be among the most influential mental health key factors. Regarding the latter aspect, studies have produced mixed findings with some of them reporting a constant improvement of mental health after the arrival (8) and some a decline (54), when after an initial “healthy migrant” effect subjective well-being decreases and adapts to that of the mainstream population. However, since nearly all of these studies have focused on the development of mental health within the first decade after migration, with regard to our present sample, expected effects might already have taken place or at least flattened out because our Vietnamese patients had already lived in Germany for an average of 16 years at the time of admission.

Finally, the migration-surrounding factors *feeling of being at home in Germany, close connection to the home country*, and the two *dimensions of acculturation* were likewise not found to be significantly associated with the current level of experienced mental distress. Concerning the latter factor, studies on the association between acculturation and psychological well-being have produced mixed findings depending on the particular mental health outcome and specific migrant population taking into consideration (55). Concerning Vietnamese migrants in Germany, one of our previous studies has reported a lower severity of depression (according to the BDI-II) among patients with both, an orientation toward the heritage and the mainstream society (12). The highest level of depressiveness, in turn, was found among patients with marginalization as acculturation strategy, who refuse to participate in both cultural communities. However, since the BDI-II focuses exclusively on the symptoms of depression, a possible explanation for not finding this connection in the current study could relate to the fact that the BSI-18 is not limited to depression, but also captures somatic complaints and symptoms of anxiety. Perhaps there is a particularly substantial overlap and clinical similarity between the appearance of depression and the characteristics of cultural detachment—insofar as, for example, social isolation can be a consequence of both states, depressiveness and cultural disengagement. Furthermore, a vicious circle would be conceivable when depression symptoms such as “loss of interest,” “withdrawal,” or “lack of motivation” additionally hamper the social exchange and thereby increase the impression of not being part of any cultural community. Consequently, more research is needed to further explore the role of acculturation by considering the depression scale independently from the other two BSI-18 dimensions.

### Limitations and Future Research Directions

Despite its notable strengths, to be one of the very few and largest clinical investigations on Vietnamese migrants in Germany and the first to address a broad range of factors that are relevant for their mental health condition, our study has several limitations that have to be mentioned: (1) The outcome was limited to self-rated symptomatology. However, self-report questionnaires may encourage respondents from socio-centric cultures such as Vietnam to answer less in consideration of social-desirability because of perceived anonymity. (2) Our study design was cross-sectional. Thus, we cannot completely rule out that there was not only an influence of the candidate variables on the GSI, but also an effect of the respective psychiatric disorder on the rating of the variables. For example, the evaluation of the financial satisfaction or the perception of discrimination could have been distorted among depressed patients by what is known as the negative attention bias or (56) negative memory bias (57). This inverse causality is a common problem in cross-sectional research and must be taken into account when interpreting our results. (3) Our naturalistic study sample consisted of predominantly female participants (76.4%). Thus, the results of the present study are not generalizable without caution to other Vietnamese populations with different gender distributions. (4) Since there is no comprehensive framework specialized in Vietnamese migrants' mental health, we had to base our preselection of candidate variables on studies with factors that are generally relevant for the mental health of all Asian migrant populations. A selection-bias can, therefore, not entirely be ruled out.

Future research on factors being associated with the mental health of Vietnamese migrants should apply a longitudinal design and include a healthy (Vietnamese) control group as well as a mixed-method (quantitative and qualitative) approach to clarify further the pathway between psychological distress and its related predictors. Another question could focus on possible interactions among the identified stressors, for instance, how a psychiatric contact impacts mental health depending on the current role of religiosity. Finally, multiple-item scales as well as external assessment instruments, should be implemented to gain further information about potential risk-factors and their effects.

## Conclusion

Out of 22 sociodemographic, clinically relevant, and migration-related candidate variables, the factors (1) *unemployment*, (2) *financial problems*, (3) *no or only little religious involvement*, (4) *no psychiatric precontact*, (5) *previous migration experiences*, and (6) *perceived discrimination* were revealed to be significantly associated with the current level of experienced psychological distress among Vietnamese migrants in Germany. Since migrants, in particular those with an Asian background, often underreport psychological complaints and underutilize mental health care services provided, the resulting population-specific risk-profile can help clinicians to identify candidates for psychiatric problems earlier and supply them with effective treatment options. Due to the cross-sectional design of the study, further research should apply a longitudinal as well as a mixed-method (quantitative and qualitative) approach to clarify further the pathway between psychological distress and its associated predictors.

## Data Availability Statement

Participants gave written informed consent to an anonymous use of the data collected for research purposes within the psychiatric outpatient clinic for Vietnamese migrants at the Charité – Universitätsmedizin, Campus Benjamin Franklin, Berlin, Germany. Therefore, following the written consensus of all participants, the de-identified raw data supporting the conclusions of this article will be made available by the authors to all researchers collaborating with the Department of Psychiatry, Charité – Universitätsmedizin Berlin, as well as upon request to those researchers with a scientific interest in using or re-using the de-identified raw data for conducting additional analyses.

## Ethics Statement

The studies involving human participants were reviewed and approved by the ethics review board of Charité – Universitätsmedizin Berlin (EA2/116/15). The patients/participants provided their written informed consent to participate in this study.

## Author Contributions

TMTT, EH, MD, AP, BH, MB, THN, VTN, and KW contributed to the study original conception and adaptation of the research design. TMTT translated all questionnaires. TMTT, EH, and MHN led most aspects concerning patients recruitment, data collection, and assessment, while THN did additional assessments of patients. MHN, SW, KB, EH, and TMTT conducted data analysis and interpretation. SW wrote the manuscript with support from TMTT, KB, KW, and EH. All authors commented and contributed to several versions until submission of the final manuscript during the review process and have seen and given final approval of the version to be published.

## Conflict of Interest

The authors declare that the research was conducted in the absence of any commercial or financial relationships that could be construed as a potential conflict of interest.
